# Metabolic resistance of the D-peptide RD2 developed for direct elimination of amyloid-β oligomers

**DOI:** 10.1038/s41598-019-41993-6

**Published:** 2019-04-05

**Authors:** Anne Elfgen, Michelle Hupert, Kevin Bochinsky, Markus Tusche, Estibaliz González de San Román Martin, Ian Gering, Silvia Sacchi, Loredano Pollegioni, Pitter F. Huesgen, Rudolf Hartmann, Beatrix Santiago-Schübel, Janine Kutzsche, Dieter Willbold

**Affiliations:** 1 Institute of Complex Systems, Structural Biochemistry (ICS-6), Research Center Jülich, 52428 Jülich, Germany; 2 Central Institute for Engineering, Electronics and Analytics (ZEA-3), Research Center Jülich, 52428 Jülich, Germany; 3 Dipartimento di Biotecnologie e Scienze della Vita, Università dell’Insubria, 21100 Varese, Italy; 4 The Protein Factory Research Center, Università dell’Insubria and Politecnico di Milano, 20100 Milano, Italy; 5 Institut für Physikalische Biologie, Heinrich-Heine-Universität Düsseldorf, 40225 Düsseldorf, Germany

## Abstract

Alzheimer’s disease (AD) is a neurodegenerative disorder leading to dementia. Aggregation of the amyloid-β peptide (Aβ) plays an important role in the disease, with Aβ oligomers representing the most toxic species. Previously, we have developed the Aβ oligomer eliminating therapeutic compound RD2 consisting solely of D-enantiomeric amino acid residues. RD2 has been described to have an oral bioavailability of more than 75% and to improve cognition in transgenic Alzheimer’s disease mouse models after oral administration. In the present study, we further examined the stability of RD2 in simulated gastrointestinal fluids, blood plasma and liver microsomes. In addition, we have examined whether RD2 is a substrate for the human D-amino acid oxidase (hDAAO). Furthermore, metabolite profiles of RD2 incubated in human, rodent and non-rodent liver microsomes were compared across species to search for human-specific metabolites that might possibly constitute a threat when applying the compound in humans. RD2 was remarkably resistant against metabolization in all investigated media and not converted by hDAAO. Moreover, RD2 did not influence the activity of any of the tested enzymes. In conclusion, the high stability and the absence of relevant human-specific metabolites support RD2 to be safe for oral administration in humans.

## Introduction

Alzheimer’s disease (AD) is a progressive neurodegenerative disorder and the most common form of dementia. Currently more than 20 million people worldwide are affected by AD and the number of patients increases continuously^1,2^. AD is an amyloid related disorder associated with misfolding and self-assembly of the amyloid-β peptide (Aβ) leading to characteristic neuritic extracellular amyloid plaques, tau aggregates and loss of neurons in the brain^3,4^. Although utterly important, preventive or curative treatments are not yet available^5^. An attractive approach for AD drug development is the intervention in the Aβ self-assembly process^5–7^ preferably reducing Aβ oligomer load as Aβ oligomers are the most neurotoxic Aβ species currently known^8–11^. One approach to target Aβ oligomer toxicity has been proposed for example by accelerating fibril formation through addition of the D-enantiomeric Aβ version and thereby reducing Aβ oligomer load^12^. In contrast, our strategy is to target Aβ oligomers directly by compound-mediated destabilization und subsequent elimination^13^. We have developed the Aβ oligomer eliminating D-peptide RD2 (all-D-peptide ptlhthnrrrrr), which improved cognition of AD transgenic mice after oral treatment^14^. Its oral bioavailability has been described to be high with a terminal half-life of approx. 60 h^15^. These promising results for oral application are probably based on its structure constituted solely of D-enantiomeric amino acid residues. It has previously been shown that the integration of D-amino acids into peptides enhances their proteolytic stability^16–23^. Furthermore, for RD2’s lead compound D3 (all-D-peptide rprtrlhthrnr), the superior resistance of all-D-enantiomeric peptides against metabolization by proteases and cytochrome P450 (CYP) enzymes contained in the gastrointestinal tract, blood and liver has recently been shown in a study by Elfgen and colleagues^24^.

**Figure 1:**
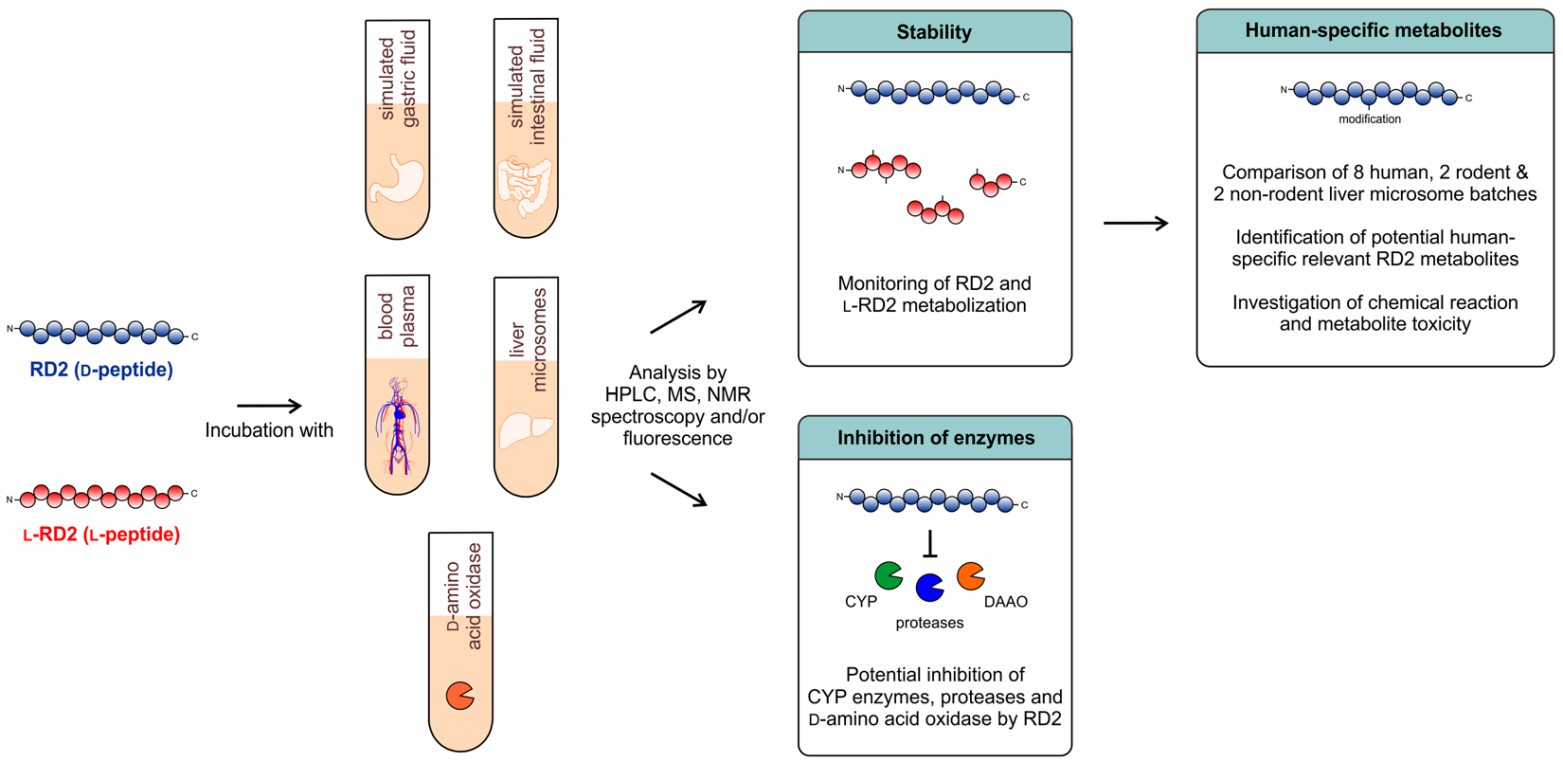
Schematic overview of the methodological approaches. It was investigated whether the D-peptide RD2 is a substrate or inhibitor for enzymes contained in simulated gastric and intestinal fluid, human blood plasma and human liver microsomes or for the D-amino acid oxidase. RD2’s mirror image l-RD2 served as comparative substance. Potential human-specific metabolites generated in liver microsomes were identified and characterized.

In this study, we determined if the advanced D-peptide RD2 is also highly resistant against metabolization by these enzymes and therefore suitable for oral application. Moreover, we investigated possible inhibitory effects of RD2 on these enzymes, including a detailed study of CYP isoforms inhibition. Additionally, activity and inhibition tests were performed with an enzyme specialized to metabolize D-enantiomeric amino acids, the human D-amino acid oxidase (hDAAO), which is mainly expressed in kidney, liver and brain^25–28^. Furthermore, it has previously been shown in preclinical toxicology studies, mandatory for the application of a phase I study in humans, that orally administered RD2 does not cause toxic effects up to high doses (complete study will be published elsewhere). Based on these findings, metabolite profiles of RD2 incubated in human liver microsomes were compared with metabolite profiles of RD2 incubated in liver microsomes of other species to identify potential human-specific metabolites and thus to forecast the safety for administration in humans^29^. Figure 1 gives an overview of the methodological approaches.

## Material and Methods

### Peptides

The D-peptide RD2 (sequence: ptlhthnrrrrr, CBL Patras, Patras, Greece) consists of 12 D-enantiomeric amino acid residues with its C-terminus being amidated. The mirror image of RD2, l-RD2 (sequence: PTLHTHNRRRRR, peptides & elephants, Potsdam, Germany), has the identical amino acid sequence but with all amino acid residues in l-configuration, also with its C-terminus being amidated. RD2 and l-RD2 have a molecular mass of 1.599 kDa. The peptide KLVFFRRRRRR (peptides & elephants, Potsdam, Germany) consists of 11 l-enantiomeric amino acid residues with its C-terminus being amidated. It has a molecular mass of 1.589 kDa.

### Media simulating the gastrointestinal tract, blood and liver

The preparation of the media was performed as described before in Elfgen *et al*.^24^. Preparation of simulated gastric and intestinal fluid (SGF & SIF) was done according to the European Pharmacopoeia 7.0. Plasma samples were obtained from human blood of a volunteer female donor. Pooled human liver microsomes were purchased from Sekisui XenoTech (Kansas City, USA; H1000, Lot: 0710494 and 1210270; H1500, Lot: 1210079), from Sigma-Aldrich (St. Louis, USA; M0692, Lot: SLBL1433V; M0567, Lot: SLBR2706V and SLBN7300V) and from Gibco, Thermo Fisher Scientific (Waltham, USA; HMMCPL, Lot: PL050; HMMCPM, Lot: PL039). Pooled Sprague Dawley rat liver microsomes were purchased from Sigma-Aldrich (St. Louis, USA; M9066, Lot: SLBN0707V) and from Gibco, Thermo Fisher Scientific (Waltham, USA; RTMCPL, Lot: RT053). Pooled cynomolgus monkey liver microsomes were purchased from Gibco, Thermo Fisher Scientific (Waltham, USA; MKMCPL, Lot: MK062) and from Sekisui XenoTech (Kansas City, USA; P2000, Lot: 1210334). The liver microsomes were diluted in an NADPH regenerating system (NRS).

### Incubation of peptides in different media and sample preparation for analysis

For the stability tests, 150 µM RD2 or l-RD2 was incubated in SGF, SIF, human plasma and human liver microsomes in triplicate at 37 °C with slight shaking for different time periods. To prevent microbial contaminations in long-term plasma incubations, 0.1% sodium azide was added to the solutions. Microsomal long-term activity was examined by incubating microsomes without peptide for up to 24 h and adding l-RD2 after 8 and 24 h. The ability to degrade l-RD2 served as indicator for their activity. For the comparison of RD2 metabolite profiles across species, 150 µM RD2 was incubated in human, rat and cynomolgus liver microsomes at 37 °C for 2 and 24 h. The peptides were extracted by precipitating the proteins with trichloroacetic acid (TCA) and subsequent centrifugation, as described previously in Elfgen *et al*.^24^. Peptides immediately extracted from the media and precipitated media without peptides served as controls.

To investigate the chemical reaction by which HSM-3 is generated, 150 µM RD2 was incubated in water with and without 30 mM formaldehyde at 37 °C for 24 h. Subsequently, the samples were treated with TCA.

The samples were analyzed by reversed phase high performance liquid chromatography (RP-HPLC) (see 2.8) or ultra high performance liquid chromatography electrospray ionization quadrupole time-of-flight mass spectrometry (UHPLC-ESI-QTOF-MS) (see 2.9).

### Inhibition assay with enzymes contained in SGF, SIF, human blood plasma and human liver microsomes

To investigate whether RD2 acts as potential inhibitor for the enzymes contained in SGF, SIF, human blood plasma and human liver microsomes, an exemplary l-peptide, KLVFFRRRRRR, was incubated in these media with and without RD2 and the potential inhibition of the l-peptide’s degradation was monitored. This control was intended to reveal a potential resistance of RD2 to degradation due to enzyme inhibition instead of pure degradation resistance. This control will not reveal RD2-based inhibition of enzymes that do not act on the substrate KLVFFRRRRRR. For this purpose, 150 µM of the l-peptide with or without 150 µM RD2 was incubated in SGF, SIF, human plasma and liver microsomes and both peptides were extracted from the media immediately or after 5 min (SGF, SIF) or 30 min (plasma, liver microsomes) with the method mentioned under point 2.3. Time dependent compound degradation or modification was followed by RP-HPLC (see 2.8).

Additionally, the potential of RD2 to inhibit the main cytochrome P450 (CYP) isoforms contained in human liver microsomes (CYP1A, CYP2C9, CYP2C19, CYP2D6, CYP3A4) was investigated in detail. The experiments were performed by Cyprotex. 0.1, 0.25, 1.0, 2.5, 10.0 and 25.0 µM RD2 in DMSO (final DMSO concentration: 0.25 to 0.3%) was incubated in human liver microsomes with 1 mM NADPH in the presence of CYP isoform-specific probe substrates at 37 °C for different time periods. Selective CYP isoform inhibitors were screened alongside RD2 as a positive control. The reactions were terminated by methanol and the generated metabolites were monitored by LC-MS/MS or fluorescence. Table 1 lists the microsome concentration, the CYP isoform-specific substrate, inhibitor and the generated metabolite as well as the incubation time used in the respective assays. All assays were performed sevenfold. For CYP1A, aliquots of the termination solutions were transferred to the relevant wells of clear bottomed 96-well plates and the formation of the metabolite was monitored by fluorescence (excitation: 535 nm; emission: 595 nm). For all other substrates, the termination plates were centrifuged at 2,500 rpm and 4 °C for 30 min and aliquots of the supernatants were combined and transferred to fresh 96-well plates. Formic acid in deionized water (final concentration 0.1%) containing internal standard was added to the supernatants prior to cassette analysis by LC-MS/MS. A decrease in the formation of the metabolite compared to the vehicle control was used to calculate the IC_50_ values.

**Table 1:** Conditions for the different CYP isoform-specific inhibition assays.

CYP isoform	Microsome concentration [mg/mL]	Specific substrate	Specific inhibitor	Incubation time [min]	Generated metabolite
CYP1A	0.25	0.5 µM ethoxyresorufin	Max. 3 µM alpha-naphthoflavone	5	resorufin
CYP2C9	1	120 µM tolbutamide	Max. 10 µM sulphaphenazole	60	4-hydroxy-tolbutamide
CYP2C19	0.5	25 µM mephenytoin	Max. 50 µM tranylcypromine	60	4-hydroxy-mephenytoin
CYP2D6	0.5	5 µM dextromethorphan	Max. 3 µM quinidine	5	dextrorphan
CYP3A4	0.1	2.5 µM midazolam	Max. 3 µM ketoconazole	5	1-hydroxy-midazolam

### Enzyme assays with the human D-amino acid oxidase (hDAAO)

For hDAAO activity and inhibition assays, the tested substances were incubated with 0.17 U/mL recombinant hDAAO, 0.1 U/mL horseradish peroxidase (Roche, Switzerland) and 4 µM flavin adenine dinucleotide (FAD) in 50 mM sodium phosphate buffer (pH 7.4). 35 µM Amplex UltraRed reagent (Invitrogen, Thermo Fisher Scientific, Waltham, USA) was added and the fluorescence signal was measured after 30 min (excitation: 535 nm; emission: 590 nm) in endpoint mode. The Amplex UltraRed reagent is a fluorogenic substrate for horseradish peroxidase that reacts with the hydrogen peroxide produced by the oxidative deamination of D-amino acids to build Amplex UltroxRed, a fluorescent reaction product, which served as indicator for hDAAO activity^2^. All enzymatic assays were conducted at room temperature in 96-well plate format using an automated liquid-handler system (epMotion 5075; Eppendorf, Hamburg, Germany). Recombinant hDAAO was overexpressed in *E. coli* and purified as previously reported^30^. The final hDAAO preparation had a specific activity of ~15 U/mg protein on D-alanine.

### hDAAO activity assay

To investigate whether RD2 acts as substrate for hDAAO, the enzyme was incubated with 2.9 mM RD2, horseradish peroxidase and FAD for up to 6 h and the fluorescence signal of the Amplex UltroxRed reagent was measured (sample values). Positive control measurements using 0.2 mM D-alanine as substrate were performed in parallel. Additionally, negative control measurements without substrates were performed. The relative emission at 590 nm was determined by subtracting control measurements to sample values.

### hDAAO inhibition assay

To examined whether RD2 inhibits hDAAO activity, the enzyme was incubated with 7.5 mM D-serine alone and together with 0 to 625 µM RD2 or sodium benzoate (a known hDAAO inhibitor)^30^, horseradish peroxidase and FAD for 30 min and the fluorescence signal of Amplex UltroxRed was recorded. hDAAO residual activity in the presence of different concentrations of RD2 or sodium benzoate was expressed relatively to the values recorded in the absence of ligands (set as 100% activity). The data were fit to a four-parameter equation to determine the curve top, bottom, IC_50_ and Hill slope.

### Cell viability assay

Rat PC12 (pheochromocytoma) cells (Leibniz Institute DSMZ, Braunschweig, Germany) were cultivated in Dulbecco’s Modified Eagle Medium (DMEM) supplemented with 10% fetal bovine serum, 5% horse serum and 1% penicillin-streptomycin. 10,000 cells per well were seeded on collagen A-coated 96-well plates (Thermo Fisher Scientific, Waltham, USA) and incubated in a 95% humidified atmosphere with 5% CO_2_ at 37 °C for 24 h. Samples with untreated RD2 and RD2 or HSM-3 purified from human liver microsomes (see 2.8) in 10 mM sodium phosphate buffer (pH 7.4) were prepared. Buffer without peptides and Triton X-100 (cytotoxic compound) served as controls. The samples were added to the PC12 cells in quadruple and incubated in 95% humidified atmosphere with 5% CO_2_ at 37 °C for 24 h. Final concentrations were 2 µM untreated RD2, purified RD2, purified HSM-3 and 0.125% Triton X-100. Cell viability was then measured using the Cell Proliferation Kit I (MTT) (Roche, Basel, Switzerland) according to the manufacturer’s instruction. The final absorbance of the formazan product was determined as the absorbance at 570 nm subtracted by the absorbance at 660 nm. All results were normalized to cells that were treated with buffer only. The experiments were performed in triplicate.

### Detection of formaldehyde in liver microsomes by derivatization with 2,4-DNPH

DNPH reacts with carbonyls (like formaldehyde) to form corresponding stable 2,4-DNPhydrazone derivatives, which can subsequently be quantified by HPLC. For derivatization of formaldehyde contained in each liver microsome batch, microsome stock solutions were mixed with 42% acetonitrile and derivatized with 0.023% (*w/v*) 2,4-dinitrophenylhydrazone (2,4-DNPH) (AppliChem, Darmstadt, Germany) diluted in 100% phosphoric acid for 3 min with slight shaking. The samples were centrifuged at 10,000 *g* and 4 °C for 5 min and the supernatant containing the derivatives was immediately analyzed by RP-HPLC (see 2.8). During the entire analysis period, background measurements of 2,4-DNPH derivatives in water were performed as formaldehyde is also contained in the atmosphere.

### RP-HPLC

The RP-HPLC system (Agilent Technologies, Santa Clara, USA; 1200 series) consisted of a manual injector, quaternary pump, a thermostatted column compartment and a variable wavelength detector. Chromatography was performed with a C18 column (Agilent Technologies, Santa Clara, USA; ZORBAX 300SB-C18 5 µm, 4.6 × 250 mm) at 25 °C and 214 nm with a flow rate of 1 mL/min. The sample injection volume was 20 µl. Chromatograms were recorded and analyzed with the Agilent software ChemStation (G2175BA; B03.01).

For sample analysis from stability tests (see 2.3), mobile phases were acetonitrile (A) and water (B) each supplemented with 0.15% trifluoroacetic acid (TFA) (*v/v*). The samples were measured isocratically at 10% solvent A for 30 min. This run conditions allowed us to detect smallest chemical changes including the differentiation between the original compound and a deamidated version (Fig. S1). RD2 and l-RD2 can potentially be deamidated at two sites, the C-terminus and the asparagine at position seven. Peak areas of the unmetabolized peptides after different incubation times were normalized to the peptides’ peak areas after direct extraction from the media. Normalized peak areas of each measurement in triplicate were averaged. Data are presented as mean ± SD. A chromatographic metabolite profile of l-RD2 in SIF was recorded with the same mobile phases but using a gradient from 0 to 30% solvent A in 30 min.

For analysis of the inhibition assay with enzymes contained in SGF, SIF, plasma and liver microsomes (see 2.4), RD2, KLVFFRRRRRR and remaining media components were separated using the mobile phases acetonitrile (A) and water (B) each supplemented with 0.15% TFA and a gradient from 0 to 50% solvent A in 15 min.

For the comparison of RD2 metabolite profiles across species (see 2.3), mobile phases were acetonitrile (A) and water (B) each supplemented with 0.1% TFA. The gradient ran from 0 to 100% solvent A and is described in detail in Table S1. The chromatographic RD2 metabolite profiles were screened for potential human-specific metabolites. RD2 and the major RD2 metabolite present after 24 h of incubation in human liver microsome batches 7 and 8, HSM-3, were purified by RP-HPLC. Mobile phases were acetonitrile (A) and water (B) each supplemented with 0.15% TFA. The samples were separated isocratically at 10% solvent A for 30 min. RD2 as well as HSM-3 were collected and the samples were freeze-dried subsequently. The amount of the purified HSM-3 was calculated based on a calibration curve of 10 to 100 µM RD2 extracted from human liver microsomes.

The formaldehyde derivatization experiments (see 2.7) were performed with a column temperature of 28 °C and a detection wavelength of 360 nm. 3 µg/mL aldehyde- and ketone-2,4-DNPH standard (CRM47651; Sigma-Aldrich, St. Louis, USA) in 50% acetonitrile was used to establish RP-HPLC conditions for separation of the formaldehyde-2,4-DNPH derivative from other ketone and aldehyde derivatives as well as unreacted 2,4-DNPH (Table S2). Mobile phases were acetonitrile (A) and water (B).

### UHPLC-ESI-QTOF-MS

The UHPLC system (Agilent Technologies, Santa Clara, USA; 1290 Infinity series) consisted of a binary pump system, an autosampler, a thermostatted column compartment and a 6250 accurate-mass QTOF-MS with an electrospray ionization (ESI) interface with a resolution of 20.000. Chromatographic separation was performed on an Acquity UPLC BEH C18 column (2.1 × 100 mm, 1.7 µm particle size; Waters, Milford, USA). Column temperature was kept at 50 °C. Flow rate was 500 µL/min. Mobile phases were acetonitrile (A) and water (B) each supplemented with 0.025% heptafluorobutyric acid (HFBA) (*v/v*) and 1% formic acid (*v/v*). Sample injection volume was 20 µL. Detection was performed with the QTOF mass detector in the ESI positive ionization mode. The mass range was set to *m/z* 100 to 1000. MassHunter software LC-MS Data Acquisition B.05.01 (Agilent Technologies, Santa Clara, CA, USA) was used to control the instrument and data acquisition.

For mass determination of a metabolite generated on incubation of l-RD2 in human liver microsomes (see 2.3), a UHPLC gradient described in Table S3 was used, which allowed the separation of RD2 from one-fold deamidated RD2 and the remaining plasma components after extraction.

For determination of HSM-3’s molecular mass, samples containing purified RD2 or purified HSM-3 (see 2.8) were diluted 1:50 in a solution composed of 85% water, 15% acetonitrile and 0.1% formic acid (*v/v/v*). The end-concentration of RD2 and HSM-3 was approx. 1 µM. Samples of RD2 incubated in water with and without formaldehyde (see 2.3) were also diluted 1:50 in this solution. The UHPLC gradient used is described in Table S4.

### ESI-FTICR-MS

To determine the accurate molecular mass and molecular formula of HSM-3, a sample containing approx. 1 mM extracted RD2 and HSM-3 each (see 2.3 for incubation and extraction conditions) was diluted 1:20 in a solution composed of 85% water, 15% acetonitrile and 0.1% formic acid (*v/v/v*) and measured with electrospray ionization Fourier transform ion cyclotron resonance mass spectrometry (ESI-FTICR-MS). Analyses were performed using a hybrid linear ion trap FTICR mass spectrometer LTQ-FT (Thermo Fisher Scientific, Waltham, USA) equipped with a 7 T superconducting magnet by infusion. The mass spectrometer was first tuned and calibrated in the positive mode following the standard optimization procedure for all voltages and settings. Mass spectra were recorded in full scan from 200 to 1000 Da with a resolution of 100.000 at *m/z* 400. All data were processed using the Xcalibur software version 2.1.

### MALDI-MS

To test whether HSM-3 contains a free primary or secondary amine, a sample containing purified HSM-3 and RD2 (see 2.8) as well as a sample with purified HSM-3 and RD2 incubated with 20 mM sodium cyanoborohydride (NaBH_3_CN) and 20 mM ^13^C- and D-labeled formaldehyde (^13^CD_2_O) at 37 °C for 3 h were analyzed by matrix-assisted laser desorption/ionization mass spectrometry (MALDI-MS). For this purpose, 1 µL of the samples was mixed with 9 µL 5 mg/mL α-cyano-4-hydroxycinnamic acid (CHCA) matrix in 50% acetonitrile with 0.1% TFA. 1 µL of this solution was spotted onto a stainless steel MALDI target (Thermo Fisher Scientific, Waltham, USA) and air-dried. Analysis was performed using a LTQ-Orbitrap XL hybrid mass spectrometer (Thermo Fisher Scientific, Waltham, USA) equipped with MALDI source (Thermo Fisher Scientific, Waltham, USA). The instrument was externally calibrated using commercial peptide standard mixtures (ProteoMass calibration kit, Sigma Aldrich, St. Louis, USA). Data were acquired in positive ion mode using the Xcalibur software v2.3 (Thermo Fisher Scientific, Waltham, USA) in a mass range from *m/z* 800 to *m/z* 2000.

### NMR spectroscopy

Approx. 30 µg purified RD2 and 60 µg purified HSM-3 (see 2.8) were separately dissolved in 200 µl D_2_O corresponding to a sample concentration of approx. 95 µM and 190 µM, respectively. All nuclear magnetic resonance (NMR) spectroscopy experiments were performed at 25 °C on a Bruker AVANCE III 750 MHz spectrometer equipped with a 5 mm triple-resonance (^1^H-^13^C/^15^N-D) TCI-cryoprobe with shielded z-gradient using 3 mm NMR-tubes. ^1^H and ^13^C resonance assignment of RD2 and the structure elucidation of the metabolite HSM-3 was achieved by two-dimensional experiments using the standard Bruker pulse-sequences HH-TOCSY, HH-ROESY, HC-HSQC and HC-HMBC.

## Results

### RD2 is remarkably resistant against enzymatic metabolization in media simulating the gastrointestinal tract, blood and liver

To investigate RD2’s resistance against enzymatic degradation, RD2 was incubated in media simulating the gastrointestinal tract, blood and liver and the unmetabolized peptide was quantified by RP-HPLC. RD2’s l-enantiomeric mirror image, l-RD2, served as comparative substance.

**Figure 2:**
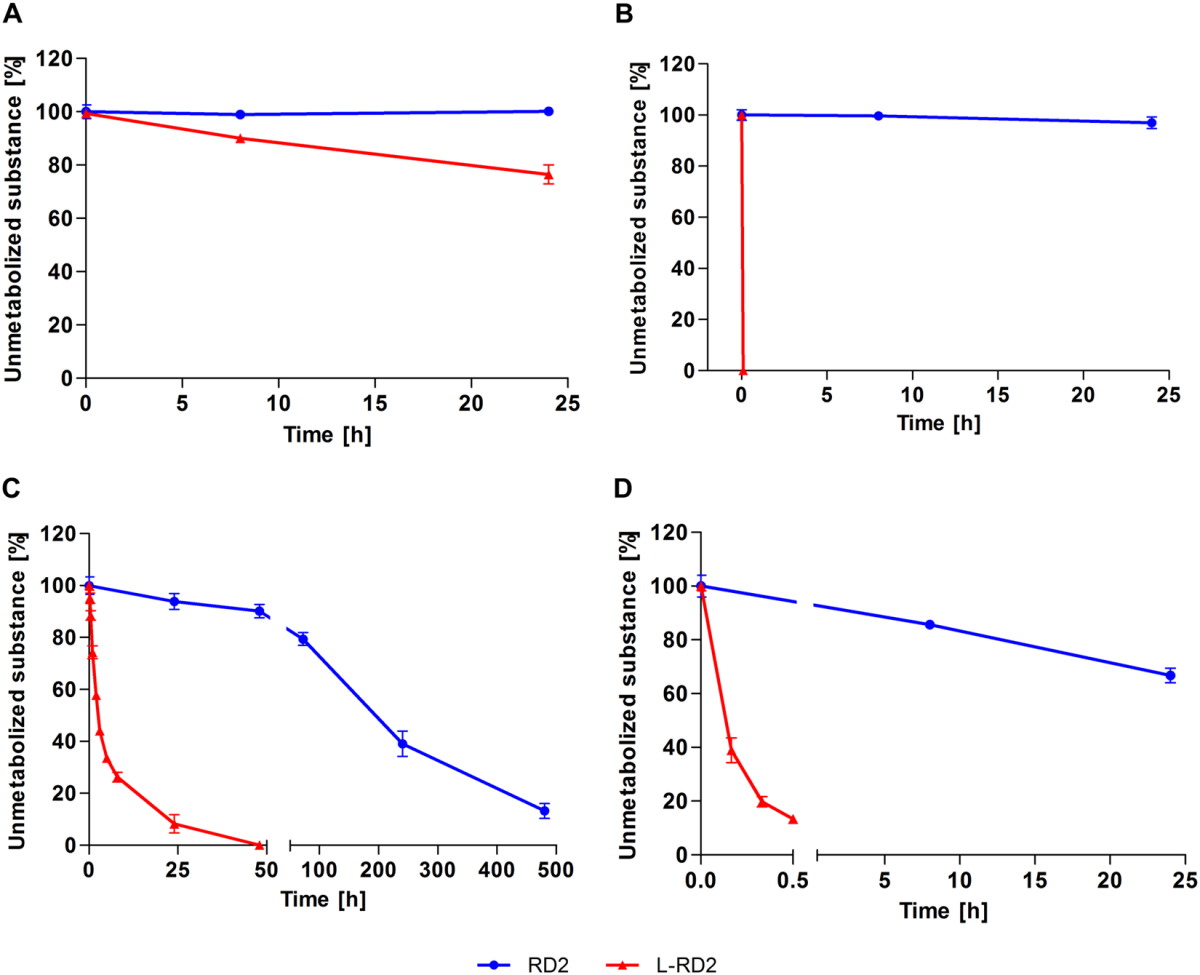
Stability of RD2 and l-RD2 in SGF, SIF, human plasma and human liver microsomes. RD2 and l-RD2 were incubated in SGF, SIF, human plasma and human liver microsomes. In SGF **(A)** and SIF **(B)**, RD2 remained stable for 24 h. In contrast, l-RD2 was degraded by 23 ± 4% within the same time in SGF and was even completely metabolized within a few seconds in SIF. Human plasma **(C)** and human liver microsomes **(D)** metabolized l-RD2 several hundred times faster than RD2. Peak areas of the unmetabolized peptides after different incubation times were normalized to the peptides’ peak areas after direct extraction from the media. Data are presented as mean ± SD (n = 3).

In simulated gastric and intestinal fluid (SGF and SIF), RD2 remained stable (≥97 ± 2%) for 24 h (Fig. 2A,B). In contrast, l-RD2 was degraded by 23 ± 4% within the same time in SGF and was even completely metabolized within a few seconds in SIF. To confirm that l-RD2 is immediately metabolized in SIF, a chromatographic metabolite profile was recorded after incubation in SIF for a few seconds (Fig. S2).

In human plasma, l-RD2 was metabolized more than 90 times faster than RD2 (Fig. 2C). In detail, 87 ± 3% of RD2 was degraded within 20 days, while 92 ± 4% of l-RD2 was already degraded within one day. The RD2 and l-RD2 data have been fitted to first order kinetics and the resulting half-lives (RD2: 216 h; l-RD2: 2 h) have been compared to each other.

In human liver microsomes, only 33 ± 3% of RD2 was metabolized within 24 h, while l-RD2 was almost completely degraded within 30 min (87 ± 1%) (Fig. 2D). A proportion of l-RD2 was increasingly metabolized to the one-fold deamidated species at one of the two possible deamidation sites within the peptide, as determined by UHPLC-ESI-QTOF-MS (Fig. S3). l-RD2 showed a mass at *m/z* of 533.6471^3+^, which is consistent with the predicted *m/z* of the three-charged ion of l-RD2 and corresponds to a monoisotopic mass of 1597.931 Da. The one-fold deamidated peptide was detected with a mass at *m/z* of 533.9752^3+^ corresponding to a monoisotopic mass of 1598.898 Da and the expected mass shift of +1 Da versus the amidated form. In contrast to l-RD2, RD2 was not deamidated in human liver microsomes. Microsomal activity was ensured during the whole experiment (Fig. S4).

### RD2 does not inhibit proteases or CYP enzymes involved in peptide metabolization

**Figure 3:**
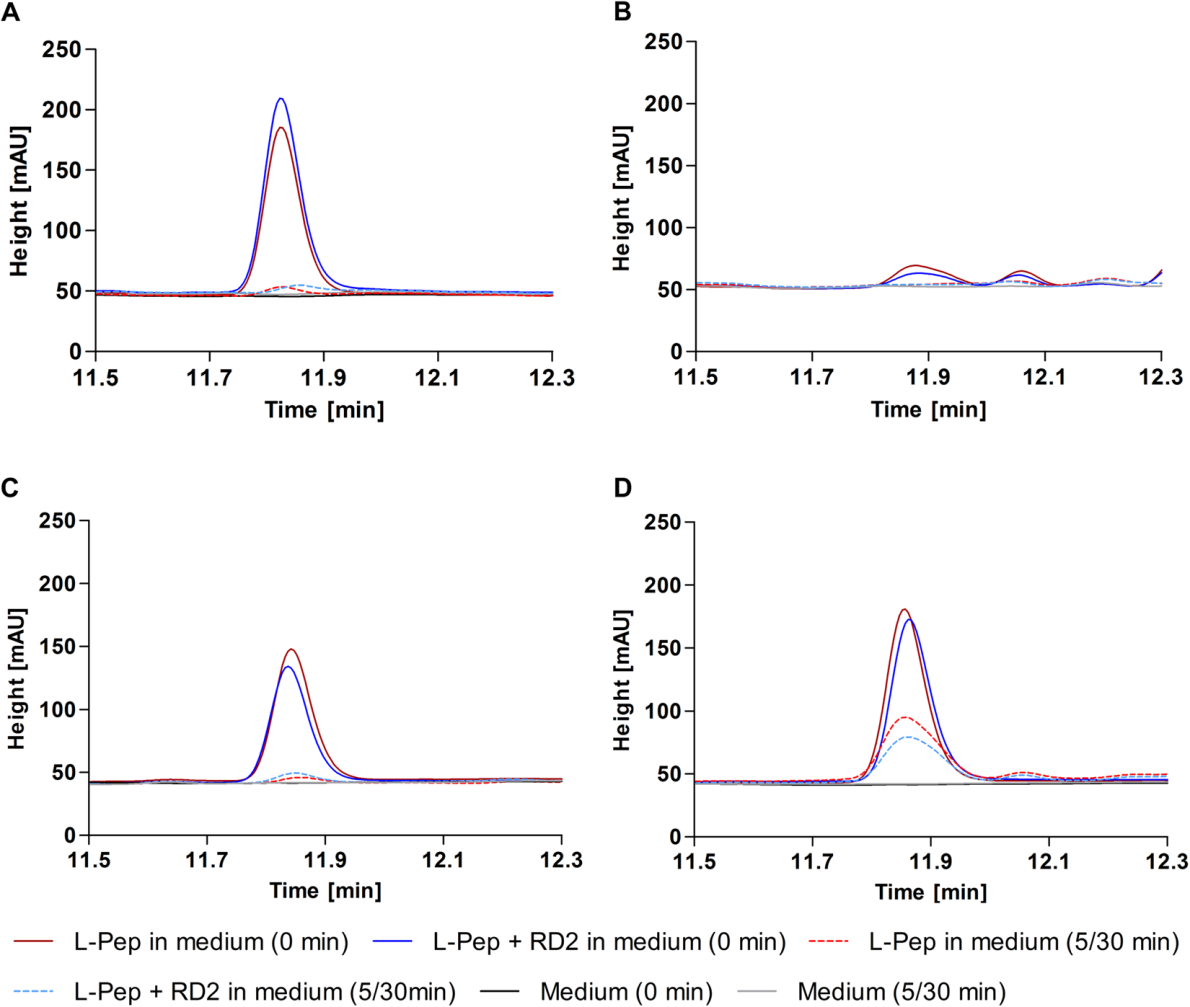
RD2 as potential inhibitor of enzymes contained in SGF, SIF, plasma and liver microsomes. Potential inhibition of the degradation of the l-peptide KLVFFRRRRRR (l-Pep) by RD2 was investigated in SGF **(A)**, SIF **(B**), human plasma **(C)** and human liver microsomes (**D**) after immediate extraction and incubation for 5 min (SGF, SIF) or 30 min (plasma, liver microsomes). SGF, SIF, plasma and liver microsomes without peptides served as controls. RD2 did not influence the degradation of the l-peptide in any of the media.

To investigate whether the high long-term stability of RD2 in SGF, SIF, human plasma and human liver microsomes is due to its ability to act as an inhibitor of one or more enzymatic activities, the influence of RD2 on the degradation of an l-peptide (KLVFFRRRRRR) was examined. The results showed that the degradation of the l-peptide was not influenced by co-incubation with RD2 in any media (Fig. 3). As proof for the rapid degradation of the l-peptide in SIF and not a failure of the extraction method, a metabolite profile of directly extracted l-peptide from SIF with and without enzymes was recorded (Fig. S5).

Moreover, we looked into detail at the potential of RD2 to inhibit the main CYP isoforms contained in human liver microsomes (CYP1A, CYP2C9, CYP2C19, CYP2D6, CYP3A4) using isoform-specific probe substrates and monitoring generated specific metabolites. Selective CYP isoform inhibitors were screened alongside RD2 as a positive control. RD2 did not inhibit any of the five CYP isoforms over the concentration range tested (0 to 25 µM) (Table S5). All of the specific inhibitors behaved as expected in the assays (Table S6)^3,31^.

### RD2 is not a substrate or inhibitor for the human D-amino acid oxidase (hDAAO)

**Figure 4:**
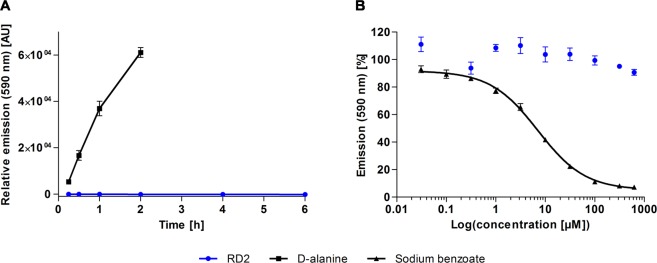
RD2 as potential substrate or inhibitor for hDAAO. The emission of a sensitive, fluorogenic reagent at 590 nm was monitored as indication for enzymatic activity. (**A**) hDAAO was incubated with RD2 or D-alanine. No change in relative emission was detected within 6 h of incubation with RD2, while a substantial increase was observed upon incubation with D-alanine within 2 h. AU: arbitrary unit. (**B**) hDAAO was incubated with D-serine alone or together with RD2 or sodium benzoate, a known hDAAO inhibitor. hDAAO activity was decreased by 92.8 ± 0.1% on incubation with 625 µM sodium benzoate, while the same concentration of RD2 only slightly affected the enzyme activity (decrease by 9.3 ± 2.1%). The IC_50_ of sodium benzoate was determined as 7 µM with a four-parameter fit.

First, it was investigated whether RD2 acts as substrate for hDAAO. For this purpose, the enzyme was incubated with 2.9 mM RD2 for up to 6 h and its activity was detected by means of a sensitive, fluorogenic reagent. No change in fluorescence emission was detected in this condition (Fig. 4A). Control measurements using 0.2 mM D-alanine as substrate were performed in parallel as a positive control. In this case, the recorded emission at 590 nm increased to 61125 ± 2120 AU upon a 2 h incubation (Fig. 4A). These observations indicate that RD2 does not act as a substrate for hDAAO.

Additionally, we examined whether RD2 acts as a hDAAO inhibitor. The enzyme was incubated with 7.5 mM D-serine alone or in the presence of RD2 or sodium benzoate (a known hDAAO inhibitor^30^) in concentrations of up to 625 µM. Enzyme activity was detected by means of a sensitive, fluorogenic reagent. While hDAAO was expectedly inactivated by sodium benzoate (a 93 ± 0.1% decrease in the activity was determined at 625 µM sodium benzoate with an estimated IC_50_ of 7 µM), RD2 only slightly affected hDAAO activity (which appeared to be decreased by 9 ± 2% at 625 µM RD2) (Fig. 4B). Thus, RD2 does not act as an effective hDAAO inhibitor.

### Identification of potential human-specific RD2 metabolites from liver microsomes

As preclinical toxicology studies with rat and cynomolguses showed that orally administered RD2 does not cause toxic effects up to high doses (complete study will be published elsewhere), RD2 was incubated *in vitro* in human (n = 8), rat (n = 2) and cynomolgus (n = 2) liver microsomes for 0, 2 and 24 h and chromatographic metabolite profiles were recorded to search for potential human-specific metabolites and hence to predict the safety for administration in humans. In six of the eight human liver microsome batches, RD2 was metabolized only slightly (human samples 1–6), while two of them showed a remarkably higher extent of RD2 metabolization (human samples 7 & 8).

**Figure 5:**
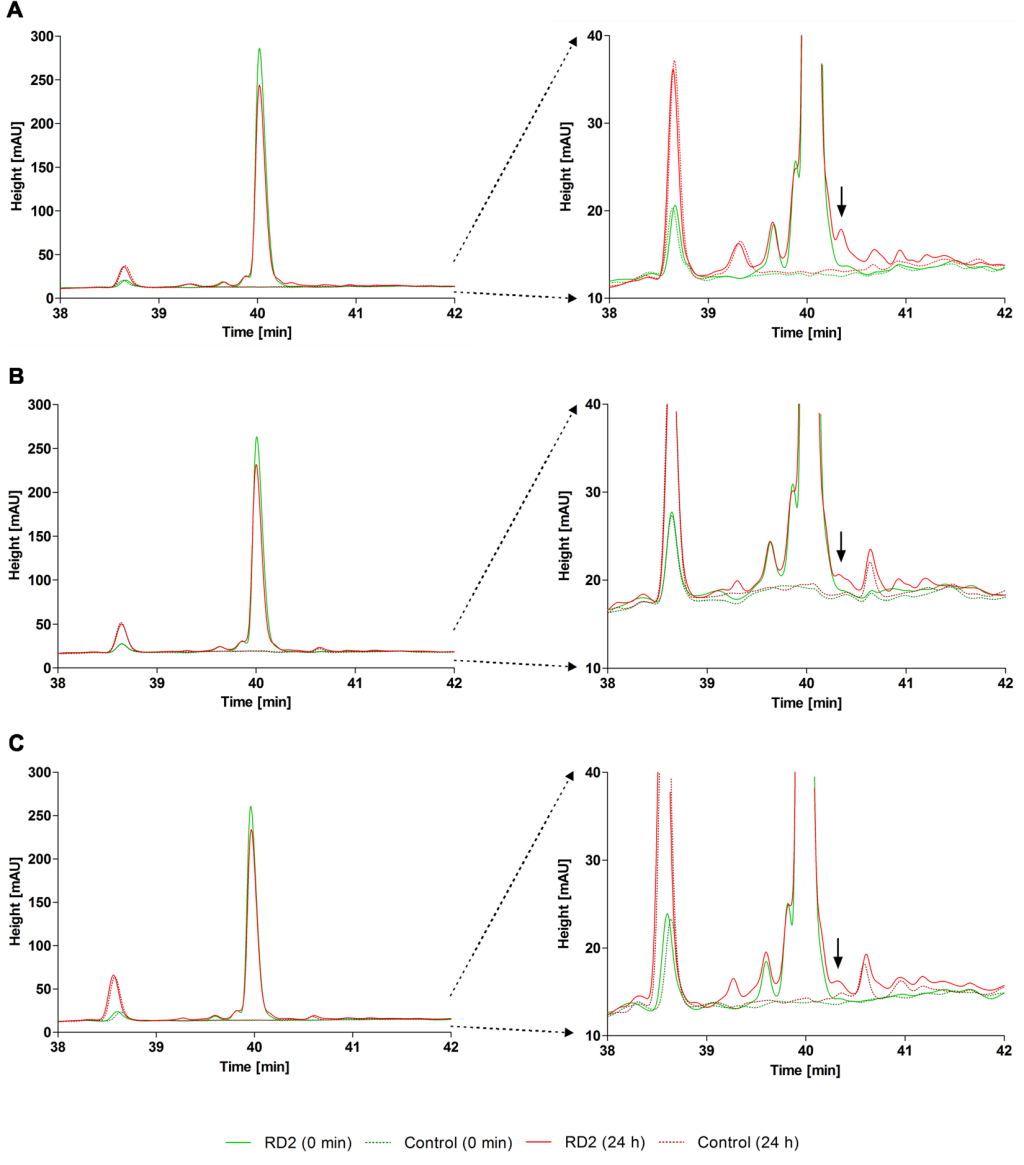
Metabolite profiles of RD2 incubated in human, rat and cynomolgus liver microsomes showing non-human-specific metabolites. RD2 was incubated in human, rat and cynomolgus liver microsomes for 0 and 24 h and chromatograms were recorded to search for potential human-specific metabolites. Incubated liver microsomes without RD2 served as controls. Only the relevant parts of the chromatogram are shown. Left: section of the chromatogram including the RD2 peak. Right: amplified section of the y-axis to focus on small metabolite peaks. In the human samples, only one metabolite with a peak area of less than 2% in comparison to the parent compound peak area appeared (**A**). This metabolite (see arrows) was also present in rat (**B**) and cynomolgus (**C**) samples.

**Figure 6:**
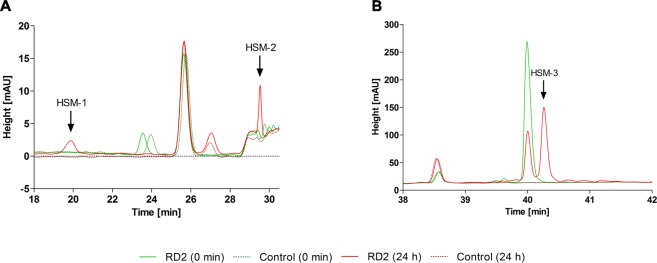
Metabolite profiles of RD2 incubated in human liver microsomes showing potential human-specific metabolites. RD2 was incubated in human, rat and cynomolgus liver microsomes for 0 and 24 h and chromatograms were recorded. Incubated liver microsomes without RD2 served as controls. Only the relevant parts of the chromatograms from the human samples are shown. Two minor potentially human-specific metabolites (HSM-1 & HSM-2) appeared in batch no. 5, which represented 3 to 5% of the parent compound (**A**). One major potentially human-specific metabolite (HSM-3), only detected in the human samples 7 & 8, represented 31 or 52% of the parent compound (**B**).

After 2 h of incubation, which is usually the maximum incubation time for more unstable substances^32,33^, RD2 appeared to be unmetabolized in the human samples 1–6 and in all rat and cynomolgus samples. To promote the metabolic degradation and increase the chance of detecting emerging metabolites, RD2 was incubated for 24 h. After extension of the incubation time, the intensity of the RD2 peak was decreased by 21 ± 7% in the human samples 1–6, by 17 ± 4% in the rat samples and by 9 ± 2% in the cynomolgus samples (Fig. 5). In all samples, the same small metabolite emerged after 24 h, whose peak area represented less than 2% of the parent compound at time point zero (incubated for only a few seconds) (Fig. 5). Consequently, this metabolite was not human-specific. Only in batch no. 5, two minor potentially human-specific metabolites (HSM-1 & HSM-2) were generated. These metabolites represented only 3 to 5% of the parent compound (Fig. 6A) and were thus irrelevant according to FDA guidelines^34^.

We became alerted of a potential human specific metabolite that could have been potentially relevant for safety but at the end has been identified as artifact from a contamination of two out of eight microsome batches already at delivery. For reasons of completeness and to avoid such unrewarded efforts in similar future studies, we briefly summarize the small side study. Only after incubation of RD2 with the human microsome batches 7 and 8, we found the RD2 peak to be already reduced after 2 h of incubation and a potentially human-specific metabolite (HSM-3) of 1 or 5% relative to the parent compound was detected (Fig. S6). After 24 h of incubation, RD2 was metabolized by 48 or 69% leading to 31 or 52% metabolite relative to the parent compound (Fig. 6B). This metabolite was not detectable after incubation of RD2 in the other six human microsome batches or with the rat and the cynomolgus microsome batches as verified by LC-MS.

### HSM-3 results from formaldehyde contamination and is not cytotoxic

The major potentially human-specific RD2 metabolite found upon incubation in liver microsomes, HSM-3, (Fig. 6B) was identified by UHPLC-ESI-QTOF-MS, ESI-FTICR-MS, MALDI-MS and NMR spectroscopy and a toxicity test was performed.

Samples containing the purified RD2 or the purified HSM-3 were analyzed with UHPLC-ESI-QTOF-MS. RD2 showed a mass at *m/z* of 533.6482^3+^, which is consistent with the predicted *m/z* of the three-charged ion of RD2 and corresponds to a monoisotopic mass of 1597.931 Da (Fig. S7). HSM-3 showed a mass at *m/z* of 537.6485^3+^. This corresponds to a monoisotopic mass of 1609.933 Da. Consequently, HSM-3 has a mass shift of +12 Da versus RD2.

**Figure 7:**
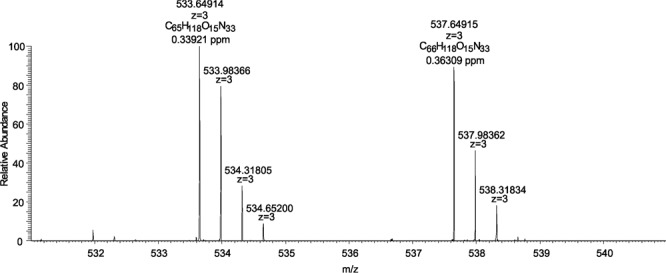
ESI-FTICR-MS analysis of RD2 and HSM-3. RD2 has a mass at *m/z* of 533.64914^3+^ and a molecular formula of [C_65_H_118_O_15_N_33_]^3+^. HSM-3 has a mass at *m/z* of 537.64915^3+^ and a molecular formula of [C_66_H_118_O_15_N_33_]^3+^.

Determination of the molecular formula of HSM-3 was performed with ESI-FTICR-MS. This measurement enables the calculation of corresponding molecular formulae based on accurate mass determination. A sample containing extracted but not purified RD2 and HSM-3 was analyzed. The measured mass of RD2 at *m/z* of 533.64914^3+^ could be attributed to the molecular composition of [**C**_**65**_H_118_O_15_N_33_]^3+^ with a deviation of 0.59 ppm (Fig. 7). For HSM-3, an accurate mass at *m/z* of 537.64915^3+^ was determined and assigned to the corresponding molecular formula of [**C**_**66**_H_118_O_15_N_33_]^3+^ with a deviation of 0.49 ppm. No other reasonable molecular formulae within the specified range (deviation < 2 ppm) were found. From this it can be concluded that the difference of 12 Da between RD2 and the metabolite HSM-3 resulted in the net addition of one carbon atom.

**Figure 8:**
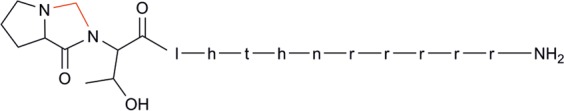
Chemical structure of the N-terminal modification of HSM-3. In comparison to RD2, HSM-3 contains an additional methylene bridge between the secondary amine of proline and the nitrogen of the peptide bond to threonine (red) generating a 4-imidazolidinone ring.

To determine the structural difference between RD2 and HSM-3, HH-TOCSY and HC-HSQC spectra were measured from each compound and signal assignment was done for protons and carbons (Table S7). By comparing the chemical shifts from both compounds, it was obvious that the proline ring was metabolized. The proline α-proton of HSM-3 shifted by 0.6 ppm upfield and the chemical shift difference from the δ-protons spread up to 0.4 ppm. The HH-ROESY spectrum of HSM-3 showed correlation signals from an additional CH_2_-group (δ_H_ 4.45 ppm, 4.35 ppm) to the α- and δ-protons of the proline ring and to the β-proton of the neighboring threonine. To validate these correlations, a HC-HMBC experiment was measured. Proton correlation signals from the additional CH_2_-group to the Cα- (64.65 ppm), Cδ- (55.00 ppm) and C=O (177.60 ppm) proline carbons and also correlations from the threonine-Hα to the CH_2_-carbon (δ_c_ 67.51 ppm) were visible. The chemical shift information together with the correlation experiments confirmed a methylene bridge between the free amino group of the N-terminal proline and the nitrogen of the peptide bond to the second amino acid residue threonine generating a 4-imidazolidinone ring. Figure 8 shows the chemical structure of the N-terminal modification of HSM-3.

The modification of RD2’s N-terminus detected by NMR spectroscopy was confirmed by high resolution MALDI-MS experiments. Reductive methylation of RD2 ([M + H]^+^ 1598.933) and HSM-3 ([M + H]^+^ 1610.933) with ^13^C- and D-labeled formaldehyde (^13^CD_2_O) and sodium cyanoborohydride resulted in a conversion of RD2 to a ^13^CD_2_H-methylated form with a mass shift of +17.033, whereas the HSM-3 peak remained unchanged (Fig. S8). This indicates that the N-terminal proline residue of RD2 is modified in HSM-3 and no longer a secondary amine. Moreover, this also indicates that the modification in HSM-3 is not a Schiff base.

The addition of only one carbon atom may be indicative of a formaldehyde reaction^35^. To test our theory, RD2 was incubated for 24 h with or without 30 mM formaldehyde in water and the samples were analyzed by UHPLC-ESI-QTOF-MS. Indeed, on incubation without formaldehyde, only RD2 could be detected, whereas on incubation with formaldehyde, HSM-3 was generated (Fig. S9). Furthermore, it could be proven by 2,4-DNPH derivatization experiments with subsequent HPLC analysis that liver microsome batches generating the metabolite contained formaldehyde, whereas microsome batches not generating the metabolite did not or substantially less (Table S8).

**Figure 9:**
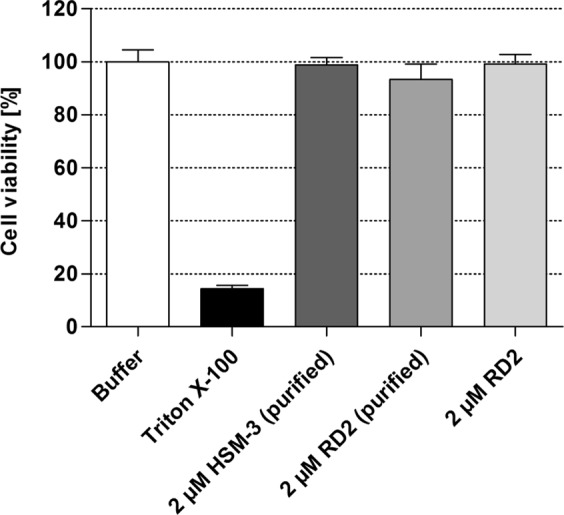
Cell viability test with HSM-3 and RD2. An MTT cell viability test with HSM-3 and RD2 purified from human liver microsomes as well as the untreated RD2 on rat PC12 cells was performed. Neither 2 µM HSM-3 nor the untreated or the purified RD2 showed any cell toxicity. A cytotoxic concentration of Triton X-100 (0.125%) was used as control.

Although it was clear that HSM-3 was only formed due to formaldehyde contamination of two of the eight human liver microsome batches, we have investigated its cytotoxic potential in an MTT cell viability assay with rat PC12 cells. 2 µM RD2 or HSM-3 purified from human liver microsomes as well as untreated RD2, which served as control for the purification step, were added to the cells and cell viability was analyzed after 24 h. Buffer without peptides and Triton X-100 (cytotoxic compound) served as controls. Neither HSM-3 (99 ± 3% cell viability) nor untreated RD2 (99 ± 4% cell viability) or the purified RD2 (93 ± 6% cell viability) showed any cell toxicity (Fig. 9). In the presence of 0.125% Triton X-100, the cell viability was reduced to 14 ± 1%.

Furthermore, high resolution MALDI-MS and UHPLC-ESI-QTOF-MS analysis of a sample of purified HSM-3 stored in solution at low temperatures revealed the presence not only of HSM-3 but also of RD2. This indicates that HSM-3 was instable and reacted back to RD2.

## Discussion

Planning oral administration of a compound implies preliminary stability testing particularly in the gastrointestinal tract as well as in blood and liver. Although compounds are increasingly metabolized upon oral administration compared to any other administration route, like intravenous or subcutaneous injection, oral administration is still the preferred administration route as it offers several advantages like low invasiveness, good patient compliance and comparatively low costs.

In this study, we demonstrate that the all-D-enantiomeric peptide RD2 is suitable for oral application as it is remarkably resistant against metabolization in simulated gastric and intestinal fluid (SGF & SIF), human plasma and human liver microsomes. Although RD2 and l-RD2 have several potential cleavage sites for proteases contained in these media^36–39^, the D-amino acid peptide bonds are less recognized. Hence, one could assume that orally administered RD2 would pass the gastrointestinal tract unmetabolized without protective formulation. This could also partially explain RD2’s long terminal half-life in blood observed previously^15^.

Furthermore, we demonstrate that RD2 is not a substrate for the human D-amino acid oxidase, an enzyme expressed in kidney, liver and brain specialized to metabolize D-amino acids^25–28^.

Additional experiments to investigate RD2’s potential to inhibit enzymes contained in the investigated media were performed, as compounds that inhibit these enzymes may cause a toxic accumulation of other substrates. Here, we demonstrate that RD2 does not act as inhibitor neither for the DAAO nor the main CYP enzyme isoforms contained in liver microsomes.

As the liver plays a major role in oral drug metabolism, it is clearly mandatory to examine the compound’s safety by screening for possible metabolites generated in the liver. Metabolites generated from metabolic biotransformation processes of phase I need particular safety evaluation. For *in vitro* assessment, usually liver microsomes are used containing phase I enzymes like the protein family cytochrome P450^40^. To assess reasonable expectations concerning metabolite induced adverse side effects in humans treated with RD2, chromatographic metabolite profiles of RD2 incubated in human, rat and cynomolgus liver microsomes were compared. We investigated the compound’s metabolism in eight different human liver microsome batches and in two different rat or cynomolgus liver microsome batches each, obtained from three different vendors in total to reveal inter-batch differences. After 24 h of incubation, three potentially human-specific metabolites were found. The two minor metabolites HSM-1 and HSM-2, found in only one of the human liver microsome batches, represented 3 to 5% of the parent compound. Additionally, the major metabolite HSM-3, present in two of the human liver microsome batches, represented 31 to 52% of the parent compound. According to current guidance^34^, we did not consider HSM-1 and HSM-2 for safety assessment but characterized HSM-3 in detail. HSM-3 was identified as RD2 with the insertion of a methylene bridge between the secondary amine of proline at position 1 and the nitrogen of the peptide bond to threonine at position 2. As described analogously in the literature^35,41,42^ the 4-imidazolidinone ring with a mass increment of 12 Da was also formed by means of a Schiff-base intermediate after adding formaldehyde to RD2 in water. As liver microsome batches generating HSM-3 were shown to contain substantially more formaldehyde than batches not generating the metabolite, we concluded that HSM-3 is generated by reaction with formaldehyde from contaminated microsome preparations. Moreover, the metabolite was non-toxic and instable and reacted back to the unmodified RD2, a process also described previously^43^.

The inter-batch differences including the identification of batches with formaldehyde contaminations revealed in the present metabolite study emphasize the importance of carefully investigating differences in metabolite formation in various liver microsome batches.

## Conclusion

In summary, we developed an all-D-enantiomeric peptide, which is a promising drug candidate for the oral treatment of Alzheimer’s disease as it is therapeutically active *in vivo* after oral administration^13,14^, has a high oral bioavailability^15^ and is highly resistant against metabolization in the gastrointestinal tract, blood and liver as well as metabolization by the hDAAO. This guarantees a high availability for absorption by the intestine even without protective formulation, increases the systemic availability, presumably contributes to the long terminal half-life of RD2 in the blood described previously^15^ and prolongs the duration of effectiveness. A high stability is advantageous as it is also accompanied by a low rate of metabolite generation and allows for a safe oral administration of the drug candidate.

## Supplementary information

Supplement

## Data Availability

All data generated or analyzed during this study are included in this published article (and its Supplementary Information files).
