# Supplementary material for: Metabolic resistance of the D-peptide RD2 developed for direct elimination of amyloid-β oligomers
Source: Sci Rep. 2019 Apr 5;9:5715. doi: 10.1038/s41598-019-41993-6 (PMC6450887; doi:10.1038/s41598-019-41993-6)
Supplement: Supplementary file 1 — Supplement [file 41598_2019_41993_MOESM1_ESM.pdf]

Supplement

Metabolic resistance of the D-peptide RD2 developed for direct elimination of amyloid-β oligomers

Anne Elfgen[a], Michelle Hupert[b], Kevin Bochinsky[a], Markus Tusche[a], Estibaliz González de San Román Martin[b], Ian Gering[a], Silvia Sacchi[c,d], Loredano Pollegioni[c,d], Pitter F. Huesgen[b], Rudolf Hartmann[a], Beatrix Santiago-Schübel[b], Janine Kutzsche[a], Dieter Willbold[a,e,*]

[a]Institute of Complex Systems, Structural Biochemistry (ICS-6), Research Center Jülich, 52428 Jülich, Germany; [b]Central Institute for Engineering, Electronics and Analytics (ZEA-3), Research Center Jülich, 52428 Jülich, Germany; [c]Dipartimento di Biotecnologie e Scienze della Vita, Università dell'Insubria, 21100 Varese, Italy; [d]The Protein Factory Research Center, Università dell'Insubria and Politecnico di Milano, 20100 Milano, Italy; [e]Institut für Physikalische Biologie, Heinrich-Heine-Universität Düsseldorf, 40225 Düsseldorf, Germany

** Corresponding author*

1. Figures

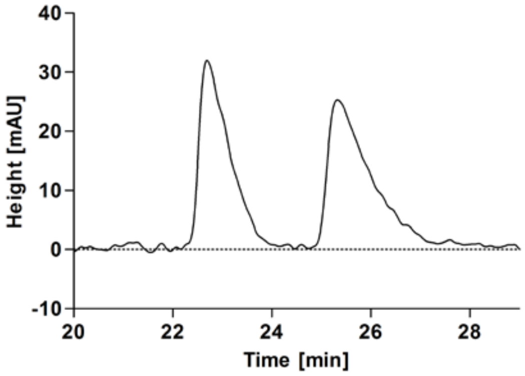

Figure S1: RP-HPLC chromatogram of separated amidated RD2 and one-fold deamidated RD2. Amidated RD2 (retention time: 22.7 min) and one-fold deamidated RD2 (retention time: 25.4) in water were separated by RP-HPLC.

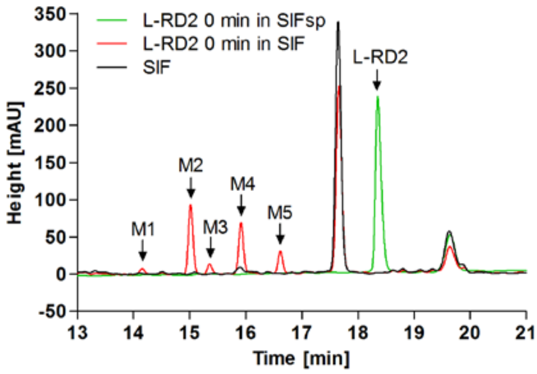

Figure S2: Metabolite profile of L-RD2 in simulated intestinal fluid (SIF). A chromatographic metabolite profile of L-RD2 was recorded after incubation in SIF for a few seconds and was compared to the profile of L-RD2 in SIF sine pancreas powder (SIFsp). L-RD2 could not be detected but several metabolites occurred (M1-5).

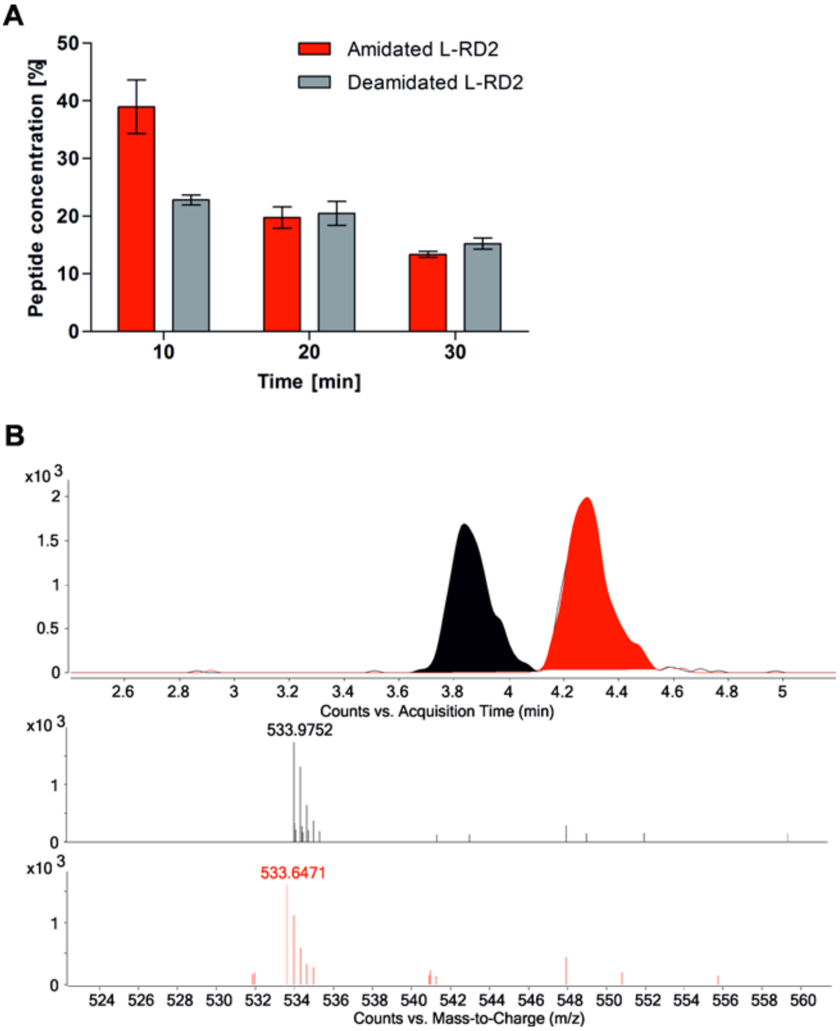

Figure S3: One-fold deamidation of L-RD2 in human liver microsomes. L-RD2 was incubated in human liver microsomes for up to 30 min. During this time, L-RD2 was metabolized increasingly to the one-fold deamidated form. **A)** RP-HPLC analysis: peak areas of the unmetabolized L-RD2 and the one-fold deamidated L-RD2 after different incubation times were normalized to L-RD2's peak areas after direct extraction from microsomes. Data are presented as mean ± SD (n = 3). **B)** UHPLC-ESI-QTOF-MS analysis of a sample with L-RD2 incubated for 10 min: extracted ion chromatogram of amidated (red) and one-fold deamidated L-RD2 (black). One-fold deamidated L-RD2 has a mass at *m/z* of 533.9756^{3+} and amidated L-RD2 has a mass at *m/z* of 533.6470^{3+}.

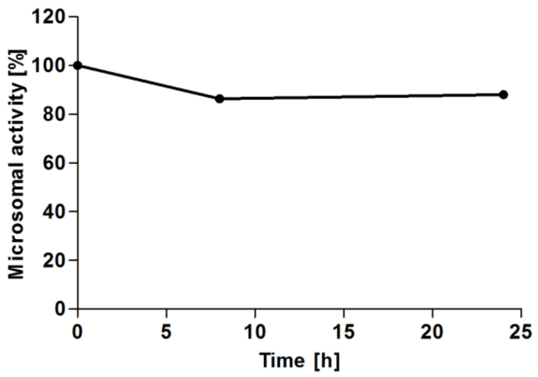

Figure S4: Microsomal activity. Microsomal long-term activity was examined by incubating microsomes without peptide for up to 24 h and examining their activity by their ability to degrade L-RD2, which was added after 8 and 24 h. The microsomes remained active during the whole experiment. Peak areas of the unmetabolized peptides after different incubation times were normalized to the peptides' peak areas after direct extraction from microsomes. Data are presented as mean ± SD (n = 3).

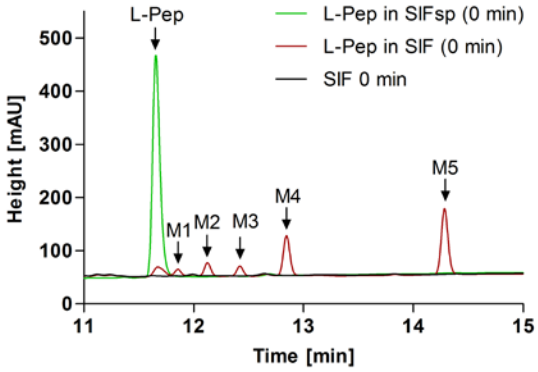

Figure S5: Metabolite profile of KLVFFRRRRRR (L-Pep) in simulated intestinal fluid (SIF). A chromatographic metabolite profile of L-Pep was recorded after incubation in SIF for a few seconds and was compared to the profile of L-Pep in SIF sine pancreas powder (SIFsp). L-Pep could not be detected but several metabolites occurred (M1-5).

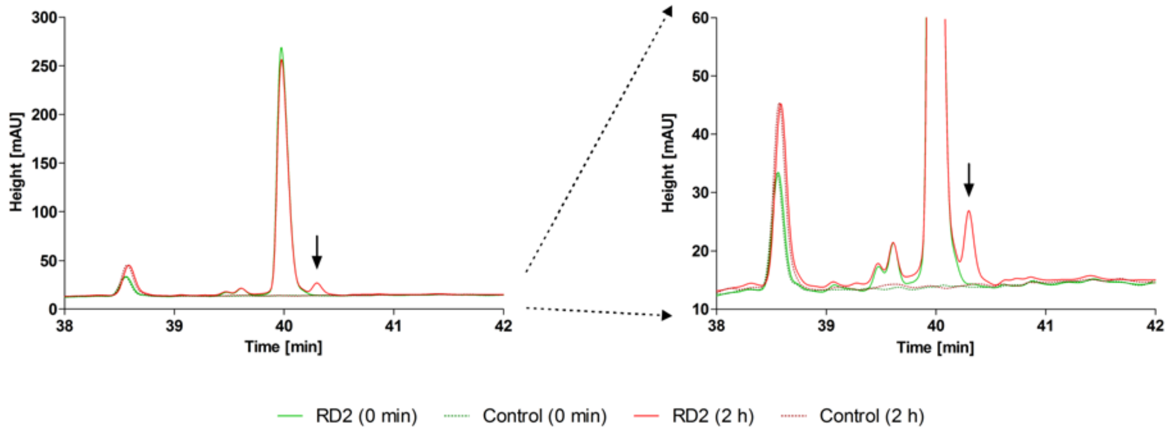

Figure S6: Metabolite profile of RD2 2 h incubated in human liver microsomes showing a potentially human-specific metabolite. RD2 was incubated in human liver microsomes for 0 and 2 h and chromatograms were recorded to search for potential human-specific metabolites. Incubated liver microsomes without RD2 served as controls. Only the relevant parts of the chromatogram are shown. One metabolite (HSM-3; arrow), only present in the human samples 7 & 8, represented 1 or 5% of the parent compound.

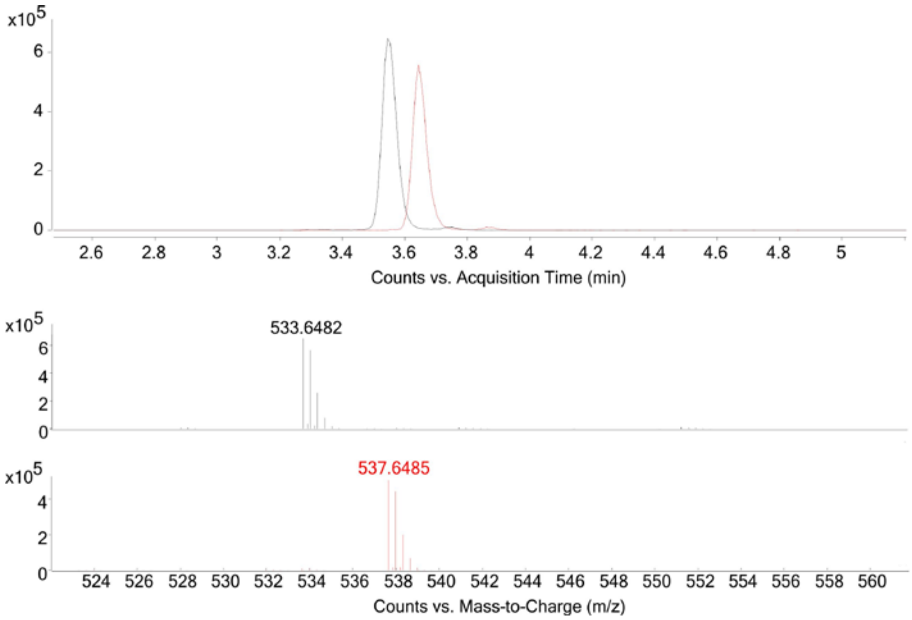

Figure S7: UHPLC-ESI-QTOF-MS analysis of RD2 and HSM-3 extracted from human liver microsomes. Overlay of RD2's (black) and HSM-3's (red) extracted ion chromatograms. RD2 has a mass at *m/z* of 533.6482^{3+} and HSM-3 has a mass at *m/z* of 537.6485^{3+}.

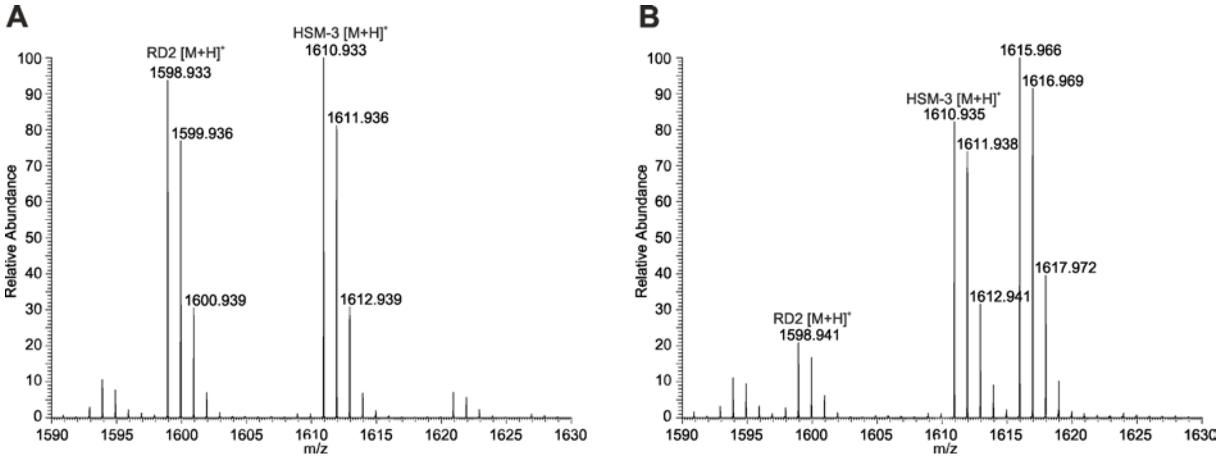

Figure S8: MALDI-MS analysis of HSM-3 and RD2. A) Analysis of the purified HSM-3 and RD2 by high resolution MALDI-MS showed a peak at *m/z* 1598.933, consistent with the predicted *m/z* of single-charged ion of RD2, and a second major peak with a mass shift of +12.000 at *m/z* 1610.933, which corresponds to HSM-3's mass. **B)** Addition of ^{13}C- and D-labeled formaldehyde ($^{13}CD_2O$) with sodium cyanoborohydride yielded a mass shift of +17.033 in the case of RD2 but not HSM-3.

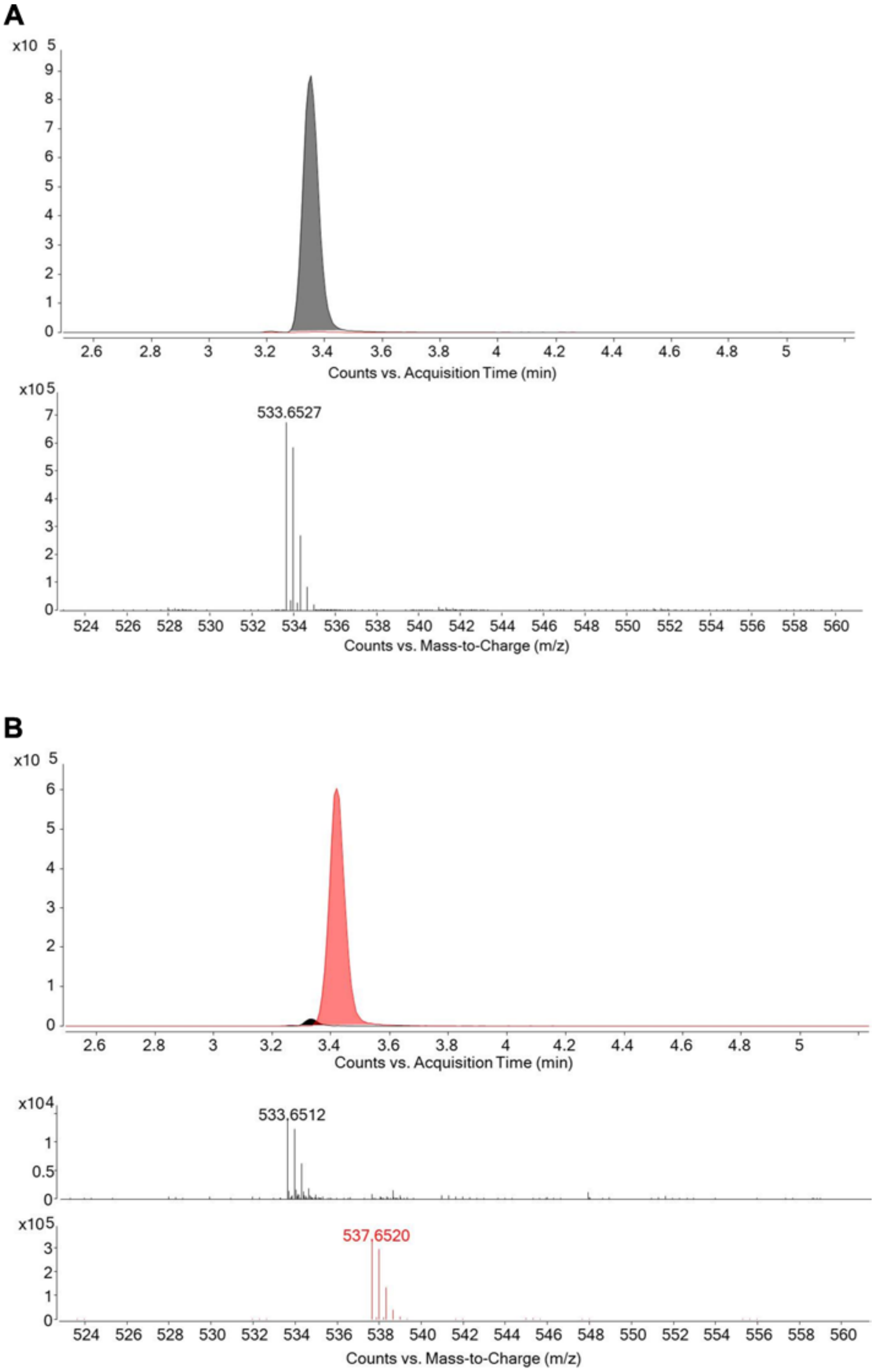

Figure S9: Reaction of RD2 with formaldehyde in water. RD2 was incubated for 24 h without **(A)** and with **(B)** 30 mM formaldehyde in water. The samples were analyzed by UHPLC-ESI-QTOF-MS. Without formaldehyde, only RD2 (black) was detected (*m/z* of 533.6527^{3+}). Under addition of formaldehyde, HSM-3 (red) was generated (*m/z* of 537.6520^{3+}).

2. Tables

Table S1: HPLC gradient used for the generation of metabolite profiles. Mobile phase A: acetonitrile with 0.1% TFA. Mobile phase B: water with 0.1% TFA.

Step	Time [min]	Mobile phase A [%]	Mobile phase B [%]
**1**	0	1	99
**2**	24	1	99
**3**	43	20	80
**4**	63	100	0

Table S2: HPLC gradient used for the separation of aldehyde- and ketone-2,4-dinitrophenylhydrazone derivatives. Mobile phase A: acetonitrile. Mobile phase B: water.

Step	Time [min]	Mobile phase A [%]	Mobile phase B [%]
**1**	0	32	68
**2**	10	32	68
**3**	15	46	54
**4**	20	46	54
**5**	25	60	40
**6**	32	80	20

Table S3: UHPLC gradient used for the separation of RD2 or L-RD2 and a one-fold deamidated peptide form. Mobile phase A: acetonitrile with 0.025% HFBA and 1% formic acid. Mobile phase B: water with 0.025% HFBA and 1% formic acid.

Step	Time [min]	Mobile phase A [%]	Mobile phase B [%]
**1**	0.0	10	90
**2**	0.5	10	90
**3**	0.6	17	83
**4**	6.0	17	83
**5**	6.6	95	5

Table S4: UHPLC gradient used for the analysis of RD2 and HSM-3. Mobile phase A: acetonitrile with 0.025% HFBA and 1% formic acid. Mobile phase B: water with 0.025% HFBA and 1% formic acid.

Step	Time [min]	Mobile phase A [%]	Mobile phase B [%]
**1**	0.0	10	90
**2**	2.0	10	90
**3**	2.1	22	78
**4**	2.6	22	78
**5**	6.0	25	75
**6**	6.1	95	5

Table S5: Inhibition of different CYP isoforms by RD2 in different concentrations.

	Inhibition [%]						
**RD2 conc.**	**0 µM**	**0.1 µM**	**0.25 µM**	**1 µM**	**2.5 µM**	**10 µM**	**25 µM**
**CYP1A**	0	0.906	6.18	9.42	1.34	-0.842	-0.198
**CYP2C9**	0	5.32	6.15	-0.871	0.607	-7.38	-26.8
**CYP2C19**	0	5.14	7.64	2.47	2.54	-5.77	-45.5
**CYP2D6**	0	7.58	1.33	0.671	3.53	-5.90	-9.57
**CYP3A4**	0	1.49	-0.223	5.77	1.71	-3.08	-12.0

Table S6: Inhibition of different CYP isoforms by CYP isoform-specific inhibitors. Determination of IC_{50} ± SD.

CYP isoform	Specific inhibitor	IC_{50} [µM]	SD [µM]
**CYP1A**	Alpha-naphthoflavone	0.0781	0.0204
**CYP2C9**	Sulfaphenazole	0.368	0.0589
**CYP2C19**	Tranylcypromine	10.7	0.654
**CYP2D6**	Quinidine	0.0451	0.00584
**CYP3A4**	Ketoconazole	0.0515	0.00381

Table S7: Signal assignment for protons and carbons after measurement of HH-TOCSY and HC-HSQC spectra of RD2 and HSM-3. RD2 und HSM-3 were measured in D_2O at 25 °C and 750 MHz.

		RD2			HSM-3		
		δ^1H [ppm]		δ^{13}C [ppm]	δ^1H [ppm]		δ^{13}C [ppm]
**Pro**	α	4.39		59.55	3.67		64.65
	β	2.40	1.96	29.84	1.99	1.80	27.10
	γ	1.98	1.94	23.61	1.67	1.62	24.50
	δ	3.37	3.33	46.51	3.03	2.54	55.00
	CO			169.83			177.60
**N-CH$_2$-N**					4.45	4.35	67.51
**Thr**	α	4.24		59.46	4.19		60.83
	β	3.99		66.94	4.03		64.52
	γ	1.07		18.80	0.99		19.38
	CO			171.07			
**Leu**	α	4.27		52.20	4.16		52.22
	β	1.51	1.41	39.85	1.40	1.31	39.48
	γ	1.47		24.25	1.36		24.32
	δ	0.82		21.90	0.73		21.94
	δ		0.76	20.79		0.66	20.60
	CO			174.08			
**His**	α	4.67		52.70	4.42		54.06
	β	3.11	3.03	26.96	2.95	2.92	
	CO			172.16			
	γC			129.85			
	δCH	7.09		117.00			
	εCH	8.22		134.38			
**Thr**	α	4.21		58.97	4.08		59.24
	β	4.08		66.94	4.01		66.78
	γ	1.07		18.80	0.95		18.64
	CO			171.50			
**His**	α	4.61		53.00	4.49		53.72
	β	3.12	3.04	27.28	2.90	2.87	
	CO			171.88			
	γC			130.28			
	δCH	7.06		116.92			
	εCH	8.11		134.55			
**Asn**	α	4.60		50.52	4.49		50.58
	β	2.73	2.66	36.02	2.66	2.59	35.73
	CO			172.35			
	γCO			174.07			
**5x Arg***	α	4.24		53.30	4.15		53.30
	β	1.77	1.70	28.20	1.69	1.61	28.20
	γ	1.57		24.40	1.48		24.40
	δ	3.13		40.50	3.04		40.50
	CO			173.40			

*All Arginine resonances were broad signals and not resolved.

Table S8: Peak areas of formaldehyde-2,4-dinitrophenylhydrazone (2,4-DNPH) in different human liver microsome batches. Batches 7 & 8 were measured twice, batches 1 - 6 once each. The background was measured three times and did not change during analysis period.

	Human liver microsome batch #			Background
	**1 - 6**	**7**	**8**	
**Formaldehyde-2,4-DNPH peak area ± SD [mAU*s]**	13.7 ± 2.2	71.7 ± 0.2	106.5 ± 1.5	8.3 ± 1.5
